# Goals in Nutrition Science 2020-2025

**DOI:** 10.3389/fnut.2020.606378

**Published:** 2021-02-09

**Authors:** Josep Bassaganya-Riera, Elliot M. Berry, Ellen E. Blaak, Barbara Burlingame, Johannes le Coutre, Willem van Eden, Ahmed El-Sohemy, J. Bruce German, Dietrich Knorr, Christophe Lacroix, Maurizio Muscaritoli, David C. Nieman, Michael Rychlik, Andrew Scholey, Mauro Serafini

**Affiliations:** ^1^Nutritional Immunology and Molecular Medicine Laboratory (NIMML) Institute, Blacksburg, VA, United States; ^2^Braun School of Public Health, Hebrew University – Hadassah Medical School, Jerusalem, Israel; ^3^Department of Human Biology, Maastricht University, Maastricht, Netherlands; ^4^School of Public Health, Massey University, Wellington, New Zealand; ^5^School of Chemical Engineering, University of New South Wales, Sydney, NSW, Australia; ^6^Department of Infectious Diseases and Immunology, Utrecht University, Utrecht, Netherlands; ^7^Department of Nutritional Sciences, University of Toronto, Toronto, ON, Canada; ^8^Department of Food Science and Technology, University of California, Davis, Davis, CA, United States; ^9^Institute of Food Technology and Chemistry, Technische Universität Berlin, Berlin, Germany; ^10^Institute of Food, Nutrition and Health, ETH Zurich, Zurich, Switzerland; ^11^Department of Translational and Precision Medicine, Sapienza University of Rome, Rome, Italy; ^12^Human Performance Laboratory, Department of Biology, Appalachian State University, Kannapolis, NC, United States; ^13^Technical University of Munich, Analytical Food Chemistry, Freising, Germany; ^14^Centre for Human Psychopharmacology, Swinburne University, Melbourne, VIC, Australia; ^15^Functional Food and Metabolic Stress Prevention Laboratory, Faculty of Biosciences and Technologies for Agriculture, Food and Environment, University of Teramo, Teramo, Italy

**Keywords:** technology, health, food system, sustainability, nutrition sciences

## Abstract

Five years ago, with the editorial board of Frontiers in Nutrition, we took a leap of faith to outline the Goals for Nutrition Science – the way we see it (1). Now, in 2020, we can put ourselves to the test and take a look back. Without a doubt we got it right with several of the key directions. To name a few, Sustainable Development Goals (SDGs) for Food and Nutrition are part of the global public agenda, and the SDGs contribute to the structuring of international science and research. Nutritional Science has become a critical element in strengthening work on the SDGs, and the development of appropriate methodologies is built on the groundwork of acquiring and analyzing big datasets. Investigation of the Human Microbiome is providing novel insight on the interrelationship between nutrition, the immune system and disease. Finally, with an advanced definition of the gut-brain-axis we are getting a glimpse into the potential for Nutrition and Brain Health. Various milestones have been achieved, and any look into the future will have to consider the lessons learned from Covid-19 and the sobering awareness about the frailty of our food systems in ensuring global food security. With a view into the coming 5 years from 2020 to 2025, the editorial board has taken a slightly different approach as compared to the previous Goals article. A mind map has been created to outline the key topics in nutrition science. Not surprisingly, when looking ahead, the majority of scientific investigation required will be in the areas of health and sustainability.

Johannes le Coutre, Field Chief Editor, Frontiers in Nutrition.

## Health

### Functional Food for Health

(Mauro Serafini)

Over the past four decades, starting from 1980 when the term “*functional food*” was coined, research on the identification and role of food to modulate key aspects of physiology and to diminish risk factors for diseases increased exponentially (2). Together with the development of research activity, the term functional food has been subject to massive communication by media, newspapers, television, and social networks, becoming widely popular to general audiences, noticeably modifying dietary habits of consumers.

According to the European Food Safety Authority (EFSA), “*functional food*” refers to “*a food, which beneficially affects one or more target functions in the body, beyond adequate nutritional effects, in a way that is relevant to either an improved state of health and well-being and/or reduction of risk of disease*.” This implies that functional foods, independently of their nutritional composition of vitamins, minerals, proteins etc., contain bioactive components that might have an impact on the modulation of systemic physiological processes, contributing to preserving human health and well-being.

Homeostasis of human metabolism and optimized resistance to stressors or exogenous threats preserves health. Thus, human nutrition and nutritional epidemiology are key in identifying functional foods and understanding which dietary patterns and nutritional habits contribute to well-being and disease prevention.

A search on PubMed, for the period from 1980 to 2009, showed that 10,208 manuscripts featuring the term “functional food” have been published, with an average of 352 papers/year. The same analysis for the period 2010-2019 reveals a massive increase in published papers on “functional food” with a total of 32,914 publications, corresponding to 3,657 papers/year, confirming the enormous interest of the scientific community in this field. However, despite the large amount of publications, the analysis reveals that only 3.9% (*n* = 396, from 1980 to 2009) and 3.0% (*n* = 975, from 2010 to 2019) of the manuscripts address clinical and randomized controlled trials. Moreover, when adding terms such as immune function, antioxidants, or inflammation, we found an equally low representation. Therefore, despite the high referencing of functional food research in the last decade, there is still limited evidence coming from human trials. It is important to follow multidisciplinary approaches, involving different experimental models from *in vitro, ex-vivo* and animals, that can provide useful insights on possible mechanisms of action. Eating, with all the consequences on health and well-being, is tightly linked to human physiology and immunity.

The efficiency of functional food depends on the processes of digestion, absorption, metabolization of the ingested molecules and bioavailability in body fluids, cells, and tissues. Moreover, it has been shown that combinations of various foods in meals can lead to a different effect than eating the single food alone, such as weakening absorption of bioactive flavonoids and reducing their antioxidant *in vivo* actions. These effects can only be revealed and confirmed in human studies because they are related to the bioavailability of the functional ingredients (3). Finally, altered efficiency of functional foods occurs with changes in patho-physiological homeostasis, such as in the presence of hypertension, low-degree chronic inflammation, insulin resistance, oxidative stress, dysbiosis etc. (4). In these pre-pathological conditions, a food is functional when it is capable of modulating biomarkers, restoring homeostatic conditions, and exerting an effective role in nutrition-related disease prevention. Under those circumstances, it is obvious how human studies, both epidemiological and interventional, represent the optimal model for understanding the functionality of a food, to ultimately enable responsible public health nutritional recommendations. Two key aspects seem to inform future directions of research on functional food.

#### Strengthening Human-Based Evidence

More research is needed involving human subjects, both in dietary intervention studies and in nutritional epidemiology. If intervention studies represent the gold standard for human research, epidemiology is a key approach in delivering observations based on large numbers of subjects. For intervention studies, it is crucial that trials be conducted on subjects likely to be more susceptible to dietary changes. Subjects characterized by ongoing risk factors for CVD, diabetes and obesity, should represent the main focus of the trials, since these individuals are characterized by alterations in physiology that predispose to overt disease. If in healthy subjects the role of functional food is to preserve homeostatic equilibrium, then in conditions of stress, it might have a role in restoring physiological conditions and/or in extending the time window before the development of the pathology. This approach would help clarify the interplay between bioactive molecules of such food and the endogenous body defenses, such as redox-network or immune system, thereby providing evidence-based recommendations for the appropriate use of food at different stages of our lives when stress conditions increase as in aging and in disease.

For nutritional epidemiology, there is need to overcome the bias related to food frequency questionnaires, 24 h recalls and dietary histories. Classic dietary assessments suffer from many biases related to the impossibility of tracking and identifying long-term dietary habits and food intake. Epidemiological studies should develop alternative and/or complementary tools to assess food intake, involving assessment of biomarkers of intake (metabolite and metabotype) or to develop smart, wearable devices able to register detailed food intake and dietary habits. When biomarkers of intake are involved, nutrition should rely on microbiology and bioinformatic fields to study the impact of functional ingredients on multiple gut-axes i.e., gut-brain, gut-urinary tract, gut-skin, and more. In the frame of the *in vivo* approach, metabolomics, or better “nutri-metabolomics,” should be used to study the real effects of the many compounds eaten with diet or bio-transformed through the gastrointestinal tract and their impact on physiology and metabolism. Nutritional sciences should evolve toward “personalized” approaches, investigate inter-individual variability to understand the basis of different “functional” responses in different subjects. The identification of clusters of subjects, grouped for their responsiveness to specific foods, will allow tailoring personalized and effective dietary advice for better disease prevention.

#### Focusing on “Real Life” Settings

Uncontrolled high intake of nutritionally unbalanced meals, characterized by excessive calorie, sugar, and fat consumption, leads to continuous postprandial metabolic stress. This, in turn, is characterized by a steep rise in risk factors such as blood pressure, insulin resistance, oxidative and inflammatory stress, triglycerides, and high glucose blood levels (5). Transforming such stressor meals into healthy meals can reduce the increase in risk factors during post-prandial processes thereby providing better protection from inflammation and oxidative stresses (6). Finding the way to decrease stress from unbalanced meals by utilizing functional foods, might represent a winning strategy for delivering to consumers simple and practical recommendations to reduce cardiovascular risk factors. Post-prandial stress should be investigated from an epidemiological approach, paying more attention to meal frequency, meal timing, and food combinations to understand if there is an advantage in consuming functional food during the meal or not. This approach can clarify the role of dietary antioxidants, such as flavonoids, characterized by a low bioavailability in body fluids, around nanomolar, but with millimolar concentration in the stomach, playing an effective role in reducing oxidative and inflammatory post-prandial stress. Finally, nutritional and antioxidant intake of subjects should consider the effect of different cooking procedures and recipes, changing the content of ingredients with respect to raw foods, to provide a more realistic picture of the effects of different dietary intakes.

### Nutrigenomics and Personalized Nutrition

(Ellen Blaak, Ahmed El-Sohemy)

The worldwide prevalence of obesity, insulin resistance (IR), and type 2 diabetes mellitus has grown dramatically since the 1980s (7). Lifestyle intervention in prediabetic individuals may reduce diabetes and metabolic syndrome risk by more than 50% in different settings worldwide despite moderate weight loss (8–10). These lifestyle programs are based on general guidelines for healthy nutrition and physical activity but tailored toward the individual lifestyle. Nevertheless, within these tailored lifestyle interventions, 30% of the participants do not respond or adhere to the intervention. Important in this respect is that it is increasingly clear that “one size does not fit all” and that personalized or subgroup- based approaches may improve intervention outcomes with respect to (cardio)metabolic health (11) and may be more effective by changing behavior (12). Although there is no unified definition of personalized nutrition, the American Nutrition Association defines it as “a field that leverages human individuality to drive nutrition strategies that prevent, manage, and treat disease and optimize health” (13). The conceptual bases, definition, strength, and limitations of personalized nutrition have been reviewed elsewhere (11–16).

Personalized nutrition may tailor dietary interventions or recommendations to one or a combination of an individual's genetic makeup, metabolic profile, microbiome, and environmental exposures. In addition, mobile apps and wearable devices facilitate real-time assessment of dietary intake and may provide feedback on physiological functions, such as blood glucose control, heart rate, or blood pressure. All the data collected from wearables, as well as from -omics methodologies, focusing on our genome, microbiome, proteome, and metabolome can be integrated with big data analytics in order to provide personalized nutritional guidance. A landmark study within this field by Zeevi et al. (17) showed that despite high interpersonal variability in post meal glucose levels, personalized diets created with the help of machine learning algorithms including dietary habits and physical activity and gut microbiota may successfully lower blood glucose responses. More recently, in the PREDICT-1 study a machine-learning model was developed that predicted both triglyceride (*r* = 0.47) and glycemic (*r* = 0.77) responses to food intake (15). The findings also showed that the heritability of post-prandial blood glucose was high (48%), which suggests a significant modifying effect of genetic variation (18). Indeed, the science of nutrigenomics aims to understand how nutritional factors interact with our genome to determine optimal health (19) as well as performance (20). There is growing recognition among researchers and clinicians that individual genetic differences play an important role in explaining differential responses to diet. Nutrients and food bioactives also alter the expression of genes and cause tissue-specific epigenetic modifications, which help explain the molecular mechanisms of gene-diet interactions (21). Novel effects of diet on health outcomes will be discovered by applying high throughput omics technologies to traditional study designs in nutritional sciences.

Sub-group-based personalized approaches may also focus on metabolic phenotype (22) or microbial phenotype (11) to develop more targeted lifestyle approaches focused on both nutrition and/or physical activity. There are indications that parameters of glucose and insulin metabolism may determine intervention outcomes with respect to body weight control and (cardio)metabolic parameters. Slightly elevated pretreatment fasting plasma glucose determined success in dietary weight loss maintenance among overweight patients on *ad libitum* diets differing in macronutrient and fiber content (23). Insulin resistance may develop in different organs, but the severity may vary between organs. Individuals with more pronounced hepatic IR have a distinct plasma metabolome and lipidome profile compared to individuals with more pronounced muscle IR (24, 25). Additionally, genes related to extracellular modeling were upregulated in abdominal subcutaneous adipose tissue in individuals with more pronounced hepatic IR, whilst genes related to inflammation, including systemic low-grade inflammation, were upregulated in individuals with primarily muscle IR (26). There are indications that these distinct IR phenotypes may also respond differentially to dietary macronutrient composition [as reviewed in (22)].

Besides genetic differences and metabolic phenotype, microbial phenotype may be of importance in personalizing the response to diet. It has been shown that specific bacterial enterotypes may predict body weight and fat mass loss on diets varying in macronutrient composition and dietary fiber (27). Furthermore, fibers or fiber mixtures that lead to a high distal colonic saccharolytic fermentation may have more pronounced effects on metabolic health (28). Notably, individuals with prediabetes may have a reduced response to diet-induced microbiota modulation with respect to host insulin sensitivity and metabolic health outcomes (11, 28).

Current dietary recommendations may be healthy for all but are based on population averages, which may not be suited for a given individual. Focusing on both individual genetic differences as well as the microbiome may represent a more targeted approach to define more personalized strategies for nutritional interventions. Evidence is needed to support the efficacy, cost-effectiveness, and additional benefits of personalized or subgroup-based approaches beyond traditional nutrition intervention approaches.

Behavior change is an important aspect of research on personalized nutrition. A number of studies have shown that disclosure of genetic information results in greater adherence and compliance to dietary recommendations (29). Individuals who receive DNA-based dietary guidance are more likely to make lasting dietary changes compared to those who receive standard dietary advice. This applies to healthy, young adults as well as older adults who are overweight or obese (30, 31). More recently, the disclosure of genetic information was shown to increase the amount of leisurely physical activity (32). Further research on the clinical impact of genetic testing for personalized nutrition is needed to advance the application and adoption of personalized nutrition technologies by consumers and healthcare practitioners.

### Nutritional Immunology

(Willem van Eden)

Interest in factors and mechanisms that contribute to balance in our immune system has increased over the past decades. This was the result of the rising incidence of inflammatory diseases, such as autoimmune diseases and allergies, especially in developed countries. In-depth exploration of cell populations and their functionality in the immune repertoire has now led to further understanding of mechanisms responsible for maintaining a healthy immune balance. The successful search for innovative immuno-therapies for cancer has uncovered the tolerance promoting pathways that frustrate effective tumor-specific immune reactivities. In a more systemic sense, tolerance promoting pathways that control unwanted immune reactivity in the case of inflammatory conditions, such as autoimmune diseases or allergies, have been revealed. It can be anticipated that, in the coming years, the latter developments will lead to a surge of new therapies aimed at reaching so-called therapeutic tolerance, leading to a permanent state of disease remission. As an adjunct to these therapies, functional foods and personalized nutrition may be expected to evolve. From various studies, some nutrients are recognized as compounds with a role in the physiological generation and maintenance of immunological tolerance. Vitamin A, which exists in plants in the form of carotenoids and in animal products as retinol, is processed into active retinoic acid (RA) by epithelial cells and intestinal dendritic cells (DCs). RA is now known to inhibit pathological Th17-mediated inflammatory responses and to promote regulatory T cells (T*reg*), which are the dedicated controllers of inflammation in the immune system (33). Although it may depend on timing of exposure and dose, Vitamin D3 is also recognized as a regulator of immune function. DCs that were treated *ex vivo* with VitD3 adopt a tolerant phenotype and are currently studied as a possible cell therapy in autoimmune diseases such as type 1 diabetes and rheumatoid arthritis (34). Preliminary clinical trials have already shown the potential of such VitD3 produced tolerant DCs in rheumatoid arthritis (35). A parallel area of research involves dietary tryptophan; its metabolites are catabolized by indolamine 2.3-dioxygenase (IDO) into kynurenines, which are involved with T cell regulation. In the gastrointestinal tract IDO is essential for induction of T*reg* by DCs (36). In addition, kynurenines signal the aryl hydrocarbon receptor (AHR), a nuclear receptor that also regulates T*reg* cells and Th17 function (37).

Also, diet may influence the immune system in an indirect manner through its impact on the composition of the microbiome. For the breakdown of indigestible dietary components such as fiber we rely on fermentation by our anaerobic colonic bacteria, to generate short-chain fatty acids (SCFA). Recently is has become evident that the SCFA are essential for the functional tuning of a variety of immune cells (38). In the case of acetate, propionate, and butyrate, for example, specific receptors on intestinal epithelial cells, such as the G protein-coupled receptors GPR41 and GPR43 have already been identified. However, their exact functional activity has remained controversial to some extent (39). Likely enough, changes in the composition of our microbiota will impact the efficiency with which SCFA are produced, and the way SCFA contribute to immune regulation. The immune system has evolved from the necessity to resist exogenous infectious invaders while maintaining tolerance for self and foreign components in our diet. The relevant processes involved are localized in our intestinal tract. In this evolutionary process, we realize that diet plays a key role by organizing oral tolerance to secure homeostasis of the immune system.

A goal for the future will be the further development of functional foods for the prevention and therapy of immune diseases. For this, deeper understanding of the exact mechanisms by which dietary components promote, or inhibit, immune-mediated disorders will remain essential. This is needed before personal guidance for food use by clinicians will become common practice.

### From Nutritional Immunology to Immunometabolism and an Accelerated Path to Cures

(Josep Bassaganya-Riera)

The immune system is a massively and dynamically interacting system with complex interactions between nutrition and metabolism that contribute to maintaining its structural requirements and delineating functionality (effector/inflammatory vs. regulatory/anti-inflammatory processes) and health outcomes. Nutrition and immunity are intimately linked from the development of the early immune system to the response to pathogens to the life-long maintenance of immune homeostasis that protects from autoimmune diseases. Immuno-metabolic mechanisms result from an integration over time of molecular and cellular networks, overlaid upon information processing representations that extend from immunological events into nutritional and metabolic processes. For instance, a balanced diet contributes to facilitating proper activation, maintenance, and regulation of the immune response through metabolic processes. Trans-disciplinary, advanced computational and data analytics methods over the past 5 years have improved our understanding of immunometabolism from mechanism to clinical applications. Multi-dimensional and high-throughput technologies have been developed to study metabolic processes in great detail and resolution, and have enabled the systematic and comprehensive understanding of complex immuno-metabolic pathways and the identification of relationships among their components in both healthy individuals and those afflicted with autoimmune diseases. However, we fail to understand how immune-metabolic changes influence the directionality of immune responsiveness. Moreover, extrapolating from molecular mechanisms at the interface between immunity, metabolism and microbiome to health outcomes and patient phenotypes remains elusive. Over the last 5 years, the integration of advanced computational methods such as artificial intelligence (AI) and mechanistic modeling with preclinical, translational, clinical research has helped bridge these gaps.

At the most fundamental level, it is well-established that different immune cell types or activation states, i.e., naïve, memory, effector or regulatory immune cells, present distinctive metabolic profiles in accordance to their immune function and energy demands. Upon initiation of the immune response, activated immune cells undergo metabolic reprogramming to meet the enhanced energetic requirements to successfully perform effector responses. Glucose uptake and anaerobic glycolysis, together with glutamine metabolism, are upregulated to generate ATP quickly but inefficiently, while oxidative pathways, including fatty acid oxidation, TCA cycle and oxidative phosphorylation are downregulated by effector cells (T*eff*) (40–42). Additionally, the increase in glycolytic rate results in the generation of a larger pool of available carbon, to be used for the subsequent new macromolecule synthesis, essential for highly proliferative and activated cells (42, 43). The critical influence of nutrient intake in host metabolism suggests that the metabolic profile of immune cells may also be altered due to changes in the diet, with direct consequences for immune cell function. These immuno-metabolic changes were strongly associated to food intake, since leptin administration restores and promotes glucose metabolism to normal levels through direct binding to the leptin receptor, resulting in production of inflammatory cytokines (44, 45). Interestingly, the effects of fasting on glucose metabolism has been reported in effector (T*eff*), but not in regulatory T cells (T*reg*) (44). This is consistent with the differential metabolic preferences in T*eff* vs. T*reg*, where T*eff* favor glycolytic metabolism and lactate production, while T*reg* maintain a more balanced metabolic profile between fatty acid oxidation and glucose consumption, that favors more efficient, but slower, ATP generation primarily from oxidative pathways (46, 47).

Metabolic modulation of immune function could provide novel immunomodulatory strategies for treating a broad range of human diseases. Identification of new immuno-metabolic hubs is key to accelerate the development of such new therapeutics. The interface of immunity and metabolism has been exploited successfully to accelerate drug development in immune-oncology and examples of successful translation into therapeutics include the PD1 blockers. In recent years, transdisciplinary studies of such processes, from the field of nutritional immunology and applied advanced computational and data analytics methods, have led to possible new therapeutic approaches in the treatment of ulcerative colitis and Crohn's disease (48–54).

The critical influence of nutrient intake in host metabolism suggests that the metabolic profile of immune cells may also be altered due to changes in the diet and food intake, with direct consequences in immune cell function. Indeed, during fasting, CD4+ T cells display lower glucose uptake and glycolysis rate (44). These changes were strongly associated to food intake, since leptin administration restores and promotes glucose metabolism to normal levels through direct binding to the leptin receptor, resulting in production of inflammatory cytokine (44, 45). Interestingly, fasting and food intake restrictions reduce inflammation and disease severity in several mouse models of autoimmune diseases (45, 55). In a mouse model of experimental autoimmune encephalomyelitis, fasting alters glucose metabolism in effector T cells, downregulates inflammatory markers and decreases overall disease severity and symptomatology (45, 56, 57). Additionally, the fasting effect on glucose metabolism has been reported in effector (Teff), but not in regulatory T cells (Treg) (44). This is consistent with the differential metabolic preferences in Teff vs. Treg, where Teff favor glycolytic metabolism and lactate production, while Treg maintain a more balanced metabolic profile between fatty acid oxidation and glucose consumption, that favors ATP generation from oxidative pathways, a lower speed, but highly efficient process (46, 47). Excessive food intake, i.e., obesity, is characterized by a chronic, low-level inflammation in T cells. Increase in adipose tissue mass is associated to upregulation of circulating adipokines, including leptin and resistin. In addition to increasing Glut1, and promoting glucose metabolism and differentiation into T cell effector subsets, leptin also induces activation of mTOR pathway, a key molecular player in regulation of T cell metabolism and effector function (44, 58). This postulates modulation of glucose metabolism in Teff cells as a key mechanism by which nutrient intake influences immune function and leverages the development of therapeutic interventions at the intersection of nutrition, metabolism, and immunity for treating metabolic and autoimmune diseases.

Metabolic responses of immune cells are critical factors in their activation, differentiation, and proliferation. Effector T cells (e.g., Th1 and Th17 cells tied to the pathogenesis of autoimmune disease) utilize aerobic glycolysis to meet the rapid, high-energy demand of cytokine production and post-transcriptional control of phenotype, thereby deriving more ATP from glycolysis (40, 59, 60). By contrast, T*reg* that are implicated in the maintenance of immune tolerance can depend on fatty acid oxidation or a combined glycolytic-lipogenic pathway and prefer deriving ATP from oxidative phosphorylation (46). Together, this suggests that nutritional or metabolic changes can be channeled as information processing systems, through central regulatory hubs at the interface of immunity and metabolism such as AKT (61), LANCL2 (62), NLRX1 (63), or PPARγ (64, 65) pathways, which can also contribute to the maintenance or disruption of a tolerogenic microenvironment.

LANCL2 is the natural receptor of abscisic acid (ABA) and a key immuno-metabolic regulator that acts as a homeostatic switch in protecting cells and tissues from inflammatory and/or metabolic distress (48, 66). Activation of LANCL2 decreases inflammation and disease severity in several models of infectious and autoimmune diseases (48, 62, 67, 68). From this immuno-metabolic program a new pipeline of LANCL2 drugs has emerged led by BT-11, an orally active, gut-restricted first-in-class therapeutic that has two investigational new drug (IND) for ulcerative colitis (UC) in May 2018 (U.S. IND 138071) and Crohn's disease in July 2018 (U.S. IND 128490). Phase I SAD/MAD trials were completed in 2018 (52) and a Phase 2 clinical trial is currently ongoing in 195 patients (NCT03861143, BT-11-201).

NLRX1 has also been identified as an immunometabolic hub with strong regulatory functions. Treatment with anti-inflammatory polyunsaturated acids, such as punicic acid (PUA) and docosahexaenoic acid (DHA), decreases NF-kB activity and inflammasome formation in an NLRX1-dependent manner. Additionally, PUA administration ameliorates inflammation via NLRX1 in a mouse model of colitis (69). Indeed, loss of NLRX1 results in increased disease severity, infiltration of pro-inflammatory populations in colonic lamina propria, and expression of pro-inflammatory mediators (67).

Both LANCL2 and NLRX1 are two novel immunometabolic hubs with multimodal mechanisms of action and potent regulatory functions; they might provide a nutritional immunology program with an advanced computational platform for target discovery. Therapeutically, targeting of central regulatory hubs in the interface of immunity, metabolism, and nutrition, is a promising path for the development of safer, and more effective therapeutics to treat infectious and autoimmune disease. These strategies rely on the modulation of the immune responses toward regulatory pathways and induction of sustained immune tolerance. Therefore, exacerbated inflammatory responses involved in the development of chronic conditions are prevented, while the ability of the immune system to fight external threats such as infection or cancer remains intact.

In summary, the Nutritional Immunology field has undergone a substantial transformation during the last 5 years. The increased presence of transdisciplinary approaches that combine advanced computational models, AI-based approaches and modeling, together with utilization of bioinformatics pipelines, and preclinical and clinical experimentation has allowed a broader exploration, facilitating the identification, prioritization, and selection of novel therapeutic targets and dissecting the molecular mechanisms downstream. These methods applied to the analysis of high-dimensional -omics (transcriptomics, metabolomics, or proteomics) and immunometabolic datasets will fuel and accelerate nutritional immunology research and discovery during the next decade.

### Performance Nutrition

(David C. Nieman)

Hippocrates averred in the fifth century BC that “*eating alone will not keep a man well, he must also take exercise. For food and exercise… work together to produce health*” (70). The human body is indeed designed for action, and health is only attained when this design is fulfilled in tandem with the proper nutritional support.

Exercise is a powerful medicine unlike any pill or nutrient, and the effective dose is only 150–300 min per week (71). The elongated muscle groups, tendons, and ligaments allow the arms and legs to engage in a wide variety of work and sport activities, while the brain coordinates delivery of blood and oxygen from the heart and lungs. The various systems of the body communicate with one another through chemical and nervous pathways to ensure a precise coordination of activity. The more these systems are used, the easier and more enjoyable exercise becomes, and the reward when repeated over time is health.

Next to training and genetic endowment, nothing is more essential to athletic performance than nutrition. Performance nutrition has a long and colorful history. Ancient Greek and Roman athletes and warriors emphasized a diet based on meat to gain the competitive edge. The legendary Greek wrestler, Milo of Crotona, reportedly consumed large amounts of meat, and was never once brought to his knees over five Olympiads (532 to 516 BC) (72). Roman gladiators believed that meat made them better warriors, a dogma that has endured to this day among athletes from many sport disciplines. Vegetarian athletes in the mid-to-late 1800s contested this belief, formed athletic and cycling clubs, and often outperformed their carnivorous competitors in long-endurance race events (73).

The modern area of performance nutrition dates to the 1960s. An early focus was on the importance of hydration, increased energy intake, and carbohydrate supplementation (74, 75). The sports nutrition research area rapidly expanded to answer questions being raised by athletes and coaches including (76–84):

1. Are the nutritional stresses imposed by heavy exertion greater than can be met by the traditional food supply? Answer: No for most athletes if they adopt healthy dietary patterns (84). The challenge for many athletes is that their dietary intake of fruits, vegetables, and whole grains falls below recommended levels.
2. Are vitamin and mineral supplements needed, and at what level of exercise training? How prevalent is iron deficiency? What type of athlete needs extra sodium and potassium? Answer: Nutrient supplements are typically not needed unless the athlete refuses to adopt a healthy dietary pattern (85, 86). True iron deficiency in athletes is rare, but can be a problem for some female athletes who have eating disorders (78). Intake of sodium and potassium is recommended during intensive and prolonged exercise bouts (77).
3. Do individuals who lift weights need protein supplements to maximize muscle size, strength, and power? Answer: Higher protein intake is recommended to support exercise-induced muscle gains, but this can typically be achieved from the traditional food supply without protein supplements (84, 85).
4. Are there performance-enhancing nutritional and herbal supplements that are beneficial, safe, and ethical? Does this list include antioxidants, caffeine, sodium bicarbonate, arginine, beta-alanine, nitrates, branched-chain amino acids, carnitine, creatine, ginseng, and β-hydroxy-β-methylbutyrate? Answer: The best evidence supports that just five supplements have performance benefits for athletes including caffeine, sodium bicarbonate, beta-alanine, nitrates, and creatine (80).
5. Are fat loading, ketogenic diets, or intermittent fasting recommended for enhanced endurance performance? Answer: No, carbohydrate is the primary fuel for the working muscle during prolonged and intensive exercise (75–78, 84, 85). Training-induced adaptations within the muscle allow a higher utilization of fats for ATP production, sparing endogenous glycogen stores for prolonged exercise.
6. Is a high polyphenol intake recommended for athletes to counter oxidative stress, inflammation, and muscle damage? Answer: Emerging evidence supports that high polyphenol intake does improve metabolic recovery from stressful exercise bouts, but more studies are needed (86–88).
7. What is the best post-exercise dietary pattern to augment metabolic recovery and training adaptations? Answer: Post-exercise intake of beverages and foods with carbohydrates, proteins, and polyphenols improve metabolic recovery and support training adaptations (80, 82, 87, 89).

Until recently, sports nutrition investigators attempted to answer these questions using a few targeted outcomes. In some studies, null results were related more to poor research designs and mismatched outcome measures than a lack of efficacy. This created confusion in the field of sports nutrition, and one solution is to shift the focus to a human systems biology approach. This paradigm shift has already started and is being driven by exponential advances in measurement technologies and bioinformatics approaches (86–92). Proteomics, metabolomics, and lipidomics provide a system-wide view of the metabolic response to exercise by simultaneously measuring and identifying large numbers of proteins, small molecule metabolites, and lipids. These outcomes provide a much better understanding of the body's response to exercise stress. And as recently reported, selected pathways for these exercise-induced perturbations in systemic biomarkers are sensitive to dietary influences (87). Moreover, multi-omics data and gut microbiome data may improve the capacity for adapting nutritional recommendations at the individual athlete level (87, 91).

The physician Galen (c 129-210 AD) was an advocate of “moderation in all things,” and accused athletes of spending their lives in “over exercising, in over-eating, and over-sleeping like pigs” (93). For most fitness enthusiasts who work out for 30 to 60 min on most days of the week, the healthy dietary pattern advocated in the 2015-2020 Dietary Guidelines for Americans is all the nutrition advice that is needed (94). A major concern for most is weight management, and this truly is achieved by moderation and a focus on long-term healthy dietary patterns.

But there is a sizeable segment of society that exercises at the high end of the exercise workload continuum. These individuals are seeking nutritional countermeasures to the physiological stresses they are experiencing. An endless number of nutritional solutions are being investigated, and the best scientific discoveries will rely on multi-omics approaches (87).

### Managing the Obesity Pandemic

(Elliot M. Berry)

To judge by the vast literature documenting the rise worldwide in the BMI of children, adolescents, and adults – particularly women and in the lower socio-economic income groups, the first decades of the twenty-first century have been a catastrophic failure in the prevention and management of obesity (95, 96). As with all “defeats,” no one has taken responsibility. Instead, there have been arguments as to whether obesity is a disease or a risk factor? (97, 98), whether it is due to nature or nurture?; or whether society or the individual is to blame? (99) Enough time has been wasted – no one, yet everyone is responsible. What is now required are concrete plans for action. These actually were set out in 2009, in admirable depth and clarity, in a landmark issue of over 300 pages (11 articles) of the *Milbank Quarterly* volume 87 number 1. Why have they not been successful? The answer is multifactorial. It is clear that no one body or organization can solve the problem, so there must be integrated national programs to tackle the increasing obesity pandemic. It is clear that the medical and public health approaches are just not good enough.

For example, a very recent Medscape presentation on “Enduring obesity – long-term strategies for a chronic disease” (100) concluded with these four points:

1. Obesity is a chronic relapsing/remitting disease that requires long-term treatment.
2. Lifestyle changes (diet and exercise) are recommended as first-line treatment in all patients.
3. Some individuals may benefit from adjunctive pharmaco-therapy if lifestyle measures are unsuccessful, and
4. Bariatric surgery may also be indicated in some patients.

These recommendations could have been given (and were in fact) over 30 years ago which implies that health professionals have just been treading water or driving with full acceleration while the gear was in neutral. That they are still current illustrates the prevention and therapeutic bankruptcy of the obesity field that does not augur well for 2025.

Therefore, a new paradigm is needed — not more of the same. Recently, nine levels of responsibilities have been identified to tackle obesity, based on a three-tier ecological model (101). Table 1 lists different levels of responsibility incorporating the sociotype ecological framework. Levels 1 – 4 represent context and systems; levels 5 – 7 society and interpersonal relationships; and levels 8 – 9 intrapersonal health and psychological make-up. At each level some appropriate action strategies are listed. Implementation of such a program will necessitate a comprehensive, coordinated, and multidisciplinary policy overseen at the national level and involving ministries of education, health, food, finance, and more.

**Table 1:** The nine multilevel responsibilities and examples of strategies required for tackling obesity [after (101)].

	**Level of responsibility**	**Some action strategies**
1	National	Prioritize the problem with focus on health inequalities and the socially disadvantaged; Health / Environment impact assessments; Legislation – “sin” taxes, sugary drinks in schools; junk food advertising
2	Food System	Equitable, sustainable food security; Food industry to reformulate products with healthier ingredients; Marketing transparency; Food labeling
3	Education System	Nutrition, cooking, activity, and lifestyle education throughout school years; Infrastructure for regular exercise; Monitoring weight and eating disorders? School lunches, Eating manners
4	Medical System	Health care system; Motivational, non-judgmental, shared-decision approach to obesity counseling; Insurance coverage; Personal example
5	Public Health	Health literacy for breast feeding, nutrition, and lifestyle choices according to social context; Positive deviance examples
6	Local Authority, Municipality	Promoting safe, leptogenic environments for all ages, especially in deprived residential areas; Farmers markets; Healthy Cities initiatives; Milan urban food policy pact
7	Society, Community	Family and communal meals; Cultural attitudes (norms, values, social support); Peer groups; Binge eating; Social media and influencers; Role models; Gender sensitivity; Civil society; Media
8	Parental	Intra-uterine effects; Breast feeding; Parenting skills; Nutrition and lifestyle education; Regulation of screen time; Personal example
9	Individual	Lifestyle choices; Self-management, self-efficacy skills; Stress management; Coping strategies; Learned behaviors; Personality; Sense of humor
		Biology: Genetics; Epigenetics; Metabolic efficiency; Endocrine status; Energy homeostasis and Brain reward neurocircuitry

At the personal level, expectations need to be realistic and distinguish between metabolic improvements (e.g., glucose, lipids, blood pressure) that occur with a loss of only 5–10% weight loss (such as from 100 to 90–95 Kg) in contrast to cosmetic improvements which may require much more. Since behavioral changes are so difficult to manage and weight loss so difficult to maintain, it is preferable to encourage activity. The reasons for this are many. Weight loss increases metabolic efficiency such that less calories are required to remain weight stable than when obese (102). Thus, after a successful weight loss it is necessary to continue to eat less. Most people do the opposite with the result that they re-gain weight. While it is thermodynamically unlikely to lose weight from exercise – one needs to walk about 100 Km to expend the calories equivalent to one Kg body weight - the health benefits are considerable since research has shown that “Fat and Fit is as healthy as Lean and Lazy,” (103) and may be even better. Chronic disease self-management programs to help people take charge should be utilized where practical (104).

In the coming years the following topics will become more prominent. Social media will determine lifestyle choices and body image characteristics through influencers, peer groups and role models (105). They will also provide a buffer to body shaming and stigmatization. But on the negative side they may encourage ignoring the medical dangers of obesity.

In order to prevent childhood obesity, more emphasis and education must be given to parents and this should be combined with school programs from kindergarten onwards. Children can become agents of change for household eating habits.

The food industry, which is part of the problem must be brought in as part of the solution to reformulate products with more healthy ingredients (106). This may be voluntary but may require positive incentives such as front-of-pack labeling or disincentives as with legislation or “sin taxes.” Such regressive taxation may be used to benefit the population for whom it is most oppressive, and revenues may go to providing parks, playgrounds, and education programs for disadvantaged children, all of which improve health outcomes. Further, junk food advertising should be banned to children.

Digital technology and the use of Artificial Intelligence will become more involved in “personalized diets and lifestyle” - from glucose and lipid and insulin (advanced sensors) monitoring (also microbiome?) to the use of personalized databases on mobile phones to help with lifestyle choices – food intake, market buying habits, cooking tips, and activity reminders.

In summary, obesity will remain the greatest global public health challenge in nutrition and lifestyle in the coming years. The pandemic needs to be tackled at all levels and the key lies in public education and political will.

### Nutrition and the Human Gut Microbiome

(Christophe Lacroix)

#### The Microbiome, an Undervalued Organ of Paramount, the Functions of Which Are Tightly Associated With Host Nutrition

Host and microbial communities have coevolved by developing a multitude of complex symbiotic interactions and synergies. The human gastrointestinal tract (GIT) harbors a dense and complex microbial community, which consists primarily of bacteria, but also archaea, fungi, viruses, and protozoa. The gut microbiota amounts to ~100 trillions bacteria, exceeds the number of host cells 1.3-fold (107), and is composed of hundreds to thousands of species, dominated by the *Bacteroidetes* and *Firmicutes* phyla (108). A large majority of the gut microbes are located in the colon, where the microbiota performs a number of essential functions for the host under the influence of diet. It has been well-approved that microbes colonizing the intestinal tract are of major importance for human health, by providing metabolic, structural, and protective functions. Over the last two decades, a multitude of still expanding research has been trying to decipher the tri-directional interactions of the diet, the gut microbiota and the host, and their impact on health and diseases.

The gut microbiota establishes during the first 2 years of life and its composition remains stable and highly individual during adulthood, each microbiome of a healthy individual containing on average several hundreds of differentially abundant bacterial species (109). However, high individuality on the taxonomic level can mask the similar core microbiome at the functional level, with coexistence of multiple distinct taxa that can perform the same focal biochemical function within each individual (108, 110). A recent study showed that unrelated subjects share only 42% of intestinal species, but 82% of the metabolic pathways (111). A large majority of microbiome studies are based on the analysis of the taxonomic composition and are mainly descriptive, therefore lacking functional and mechanistic insights and causal relationship. The high individuality of the gut microbiome at taxonomic levels does not necessarily translate in different responses to the diet and more focus in research could be placed on conserved functions and metabolites.

With 3.3 million unique microbial genes, compared to about 20'000 genes of the human genome, the gut microbiota exhibits tremendous functional potential (112). The human gut microbiota's functional capacity equals that of the liver and has been considered as “a virtual organ within an organ” (113). The different beneficial functions of the human gut microbiota can be categorized as metabolic [e.g., short-chain fatty acid (SCFA) production], structural (including immune system development, epithelial barrier enhancement), and protective (e.g., pathogen displacement and immune priming). Certain microbiota profiles, referred to as dysbiotic microbiota, have been associated with detrimental effects for the host. Thanks to recent next-generation sequencing technologies (16S rRNA sequencing for composition profile, and shotgun metagenome sequencing revealing the metabolic potential), consistent signatures in the gut microbiome of patients with microbial dysbiosis-associated disorders were identified. In particular, decrease in taxa diversity, decreased activity of the *Lachnopiraceae* and *Ruminococcacea* families, which include butyrate-producers were reported (114, 115). The SCFA butyrate acts as primary energy source for intestinal epithelial cells and has immune modulatory effects (116). Further omics methodologies have been used to measure gene expression (transcriptomics), and the resulting pool of metabolites (117).

However, so far, there is still limited understanding in the causal relationship between the human gut microbiota and dysbiosis-related disorders. It is expected that ongoing and future research will strengthen the mechanistic understanding of the role of microbes in onset, progression and treatment of dysbiosis-related disorders (118). The focus of microbiome research will be on the development and application of efficient strategies to prevent and revert microbial dysbiosis and cure with diet being a major driver chronic microbiota-associated disorders (119).

#### Diet Is the Main Modulator of the Gut Microbiome

The gut microbiota has emerged as a key player in the development and maintenance of human health, and perturbations have been associated with various physical and mental disorders (120–122). Among major factors driving an ecosystem composition and function are the host diet providing nutrients that feed the microbes, allowing microbial growth and metabolism and supporting robust microbe-microbe and microbe-host interactions. Diet is therefore expected to impact tremendously on the composition and functionality of the microbiome. The amount and quality of undigested food components reaching the colon is highly dependent on the diet (amount of macronutrients and micronutrients, and especially fibers), the upper digestive capacity, and the food matrix characteristics also impacted by processing. In exchange, the microbes supply calories (estimated to about 10% of total calories in the diet), and various nutrients for the host (such as short chain fatty acids, amino-acids, vitamins, etc.), and metabolites involved in crosstalk and regulation of host physiology. It is, therefore, crucial to better understand the relationships between the diet, the gut microbiota and the host, starting by investigating the specific effects of the main components of the diet. Recent research has shown the potential of combining different levels of complexity, *in vitro* and *in vivo* within a coherent strategy, to decipher functions and mechanisms of food components. In the future, the purpose of food should both sustain the host nutritional requirements and consider the effects on the gut microbiota, promoting a physiological and healthy balance.

#### Models Are Key for Mechanistic Studies on How Nutritional Factors May Affect the Gut Microbiota

Experimental *in vivo* human cohort studies remain the gold standard for nutrition and gut microbiome research. However, human studies are strongly limited by their complexity, costs, difficult control, and sampling (mainly limited to feces for the gut microbiota) (123). Furthermore, human studies are mainly restricted to descriptive results since they seldom have the degree of control over environmental factors required for mechanistic hypotheses. Therefore, in human microbiome studies the dominance of correlation-based vs. causation-based results still remains an important limitation. The use of animal and *in vitro* models has gained momentum in recent years as a powerful strategy to investigate factors influencing the gut microbiota and test hypotheses. Investigations of gut microbiota/host interactions have extensively been performed in controlled animal studies carried out with conventional and gnotobiotic rodents (mainly mice and rats) to study the impact of diet and specific food components on the rodent or human gut microbiota, respectively. Extrapolation of data to the human host should be done with care considering the different physiology, microbiota and for gnotobiotic animals, the “unnatural status” of animals (124, 125). One major trend in the near future is the necessity to replace, reduce, and refine animal experiments (3R principle). This surely has already stimulated the use, and further development, of *in vitro* models for the human and animal gut.

*In vitro* fermentation models are particularly well-suited for screening dietary factors for possible functions in highly controlled settings and before moving on to *in vivo* investigation of effective conditions (123, 126). This trend is likely to continue or even expand with the development of new models, owing to societal and ethical considerations. For example, new promising models combining fermentation and cellular compartments have been proposed, for example biomimetic human gut-on-a-chip models (127, 128). However, before applying *in vitro* models in gut research, great care should be paid to the selection of suitable models and conditions, considering unique features (from simple anaerobic batch culture systems in flasks to multistage continuous flow models) but also their limitations with respect to the scientific questions under study. Models are only representations of reality with high simplification (123). Therefore, the limits of the models and obtained data may directly affect the internal and external validity of the research. These aspects of modeling are often overlooked in designing and publishing research, and many claims based on published model data are not always supported by valid assumptions.

The combination of *in vitro* and animal models with human studies offers great potential for advancing knowledge on gut microbiota in health and disease. Despite the difficulties and challenges, investigating the role of the microbiota in humans, proofs of concept and mechanistic validation must be performed ultimately in well-designed human studies. The application of multiscale strategies integrating valid *in vitro* and animal models and human observations combined with modern omics technologies are required to elucidate hypothesis-based mechanistic levels and understand the roles of diet and the gut microbiota in health and disease.

#### Carbohydrate Metabolism Is Dominant but There Are Other Nutritional Targets for the Modulation of the Gut Microbiota Functions

The most dominant pathways of the human gut microbiome belong to carbohydrate metabolism, which is the primary source of energy for the bacteria and metabolite generation. Fermentation of the carbohydrate fraction of the diet in the gut involves a complex trophic chain, starting with complex polysaccharides containing bonds that are resistant to host digestive enzymes and ending with the synthesis of SCFA and the production of different amounts of gases (129). Reduced microbial diversity, fermentation capacity, and SCFA production in fecal samples of large clinical cohorts have been associated with several chronic diseases, emphasizing the importance of a diet rich in non-digestible fibers. It is therefore not surprising that during the past two decades, a major focus of nutritional research associated with the gut microbiota has been on carbohydrate fermentation (fibers, prebiotics, and FODMAP), with opportunities for prevention and treatment of obesity, diabetes, and other related metabolic disorders through manipulation of the gut ecosystem.

On the other hand, there is a multitude of other research opportunities linking food products and components to host health via microbiome modulation. Several important nutrients for both microbes and the host are attracting increasing interest for research and as potential food modulators for the human gut microbiome. Some of these nutrients may be highly active on microbes at very low dosage compare to fibers that can induce gas overproduction and bloating. Such nutrients include micronutrients (minerals such as iron, selenium, zinc, and different B-vitamins). Addition of micronutrients to foods, eventually combined with encapsulation for gastric and small intestine protection, is possible without negative impact on flavor and texture or heavy reformulation and process adjustment.

Each day, between 3 and 15 g of dietary proteins and peptides escape digestion in the small intestine and reach the colon. Proteins provide an important source of nutrients for bacteria and a substrate for the production of several beneficial or harmful metabolites (130). For example, proteins can be digested through dissimilatory sulfate reduction by commensal bacteria leading to hydrogen sulfide. H_2_S and other products such as phenols, indoles and heterocyclic amines from aromatic amino acids may have high toxicity to colonocytes and have been implicated in IBD and colorectal cancer (131). The development and consumption of highly processed plant proteins that closely imitate traditional meat products is increasing worldwide. Compared to animal-derived protein sources, plant proteins generally exhibit a low digestibility (132). Harsh process conditions (heating, pressure, extrusion) together with the formulation with polysaccharides and binding agents for texture and consistency can change protein digestibility and the supply of protein to the colon. In addition to sustainability, research is urgently needed to evaluate formulation- and processing-induced nutritional effects of plant proteins, considering both macronutrient delivery to the host, but also effects induced on the microbiota resulting from change in matrix and digestibility.

Within the field of probiotics, there is still a clear need to better link microbes to their effects and mechanisms and provide strong efficacy data on otherwise healthy populations. An area of particular interest has emerged recently in the probiotic field, connecting the brain and the gut microbiota. This field of application is probably one of the most intriguing area in microbiome research, opening a new paradigm for improving the quality of life with societal impacts (122). While the importance of commensal intestinal microbiota for optimal gut-brain function is no longer disputed (121, 133), the exact underlying mechanisms of communication between the microbiome and the brain are still largely unknown. Recent analyses of large human cohorts have provided further evidences of the neuroactive potential of the gut microbiome and its association with neuropsychiatric conditions, such as mental quality of life and depression (122). Various metabolites with neuroactive potential (generally derived from amino acids), such as tryptamine, serotonin, dopamine, or γ-aminobutyric acid (GABA), are naturally produced by microbes in the gut. For example, *Lactobacillus* and *Bifidobacterium* strains can synthesize significant amount of GABA and their administration to mice and rats resulted in decreased depressive behavior, reduction of corticosterone-induced stress and anxiety, and reduced visceral pain sensation (134, 135). The butyrate-producing genera *Faecalibacterium* and *Coprococcus* were consistently associated with higher quality of life, while *Dialister* and *Coprococcus* were shown to be depleted in depressive patients (122). It is likely that research on probiotics preventing or acting on behavior and psychiatric diseases will rapidly develop in the next few years, given clear potential mechanistic hypotheses and biomarkers for testing.

### Nutrition and the Brain

(Andrew Scholey)

As for other areas of nutrition science, the impact of nutrition on brain function has seen enormous growth over the past few decades. Indeed, the brain is sensitive to nutritional changes and the effects of diet. This is partly due to its functional properties. Despite comprising around two percent of an adult human's mass, the brain is responsible for upwards of 20 percent of basal metabolism (136). This makes neural tissue particularly susceptible to damage from oxidative stress and inflammation. The brain also has limited glial glycogen stores, which are readily depleted (e.g., during an overnight fast, making brain activity sensitive to more immediate fluctuations in blood flow and glucose metabolism). Indeed the discovery of the differential distribution of central glucose transporters (GLUTs) (137) and of transport mechanisms for insulin (138) changed the view of the nutrition-brain axis.

Clearly, many neuroactive substances originate from ingestible sources. Indeed most, if not all, psychoactive drugs have edible botanical sources. These include components of herbs and spices, and also certain foods. Many psychoactive foods contain caffeine and it has been suggested that this may be wholly responsible for their beneficial effects on cognition and particularly alertness and attention. But this appears not to be the case. Firstly, caffeine-rich botanicals often contain other physiologically active compounds. Certain of these, such as the catechins (found in tea), chlorogenic acids (coffee), resveratrol (grape), and cocoa flavanols are “vasoactive” [e.g., (139, 140)], resulting in vasodilation and/or increasing systemic and central blood flow. This represents a physiologically plausible means of improving brain function.

Acute effects of nutrition on the brain have partly evolved from studies of the effects of simple fats and carbohydrates. These include administration of glucose and other carbohydrates. Glucose loading reliably improves cognitive function during conditions of mental demand (141, 142) and this appears not to be a simple replenishment of overnight fasting-related deficits (143). Clearly, administering glucose at the levels which result in cognitive enhancement (typically 25 to 50 g) is not a realistic nutritional strategy but it does provide a useful model system for probing acute cognitive effects of macronutrients. Indeed, glucose loading has been used in some of the few neuroimaging studies of nutritional interventions both on appetite signaling (144) and cognition enhancement (145). These have gone beyond examination of glucose effects on specific neuroanatomical loci during activation tasks to include studies of changes in connectivity during appetite signaling (146) and cognitive functioning (147). The application of neuroimaging to understand central effects of human nutrition has revealed plasma nutrient biomarker patterns, which predict central neural network efficiency and cognitive abilities (148). This work has heralded in a new era coined “nutritional cognitive neuroscience” (149). Future work in this area will adapt methods of pharmaco-imaging to apply them to “nutraimaging.” There are already promising results from small trials applying magnetoencephalography (MEG) to understand the anti-stress effects of the tea amino acid theanine (150).

Beyond day-to-day fluctuations in mood and neurocognitive function, another small human trial has reported increases in a marker of adult neurogenesis following 12-weeks cocoa flavanol administration (151). Turning to longer-term interventions, there has been much attention on the role of nutrition in neurodevelopment (152) and brain aging (153), including neuroprotection (154). Regarding neurosenescence, the 2017 Lancet commission on dementia (155) identified nine potentially modifiable risk factors for dementia. These include several, which are affected by nutrition directly (midlife obesity and hypertension, late life diabetes) and indirectly (late life depression and physical inactivity).

Epidemiological and cohort studies confirm that so-called “prudent” diets – e.g., the Mediterranean diet - appear to impart neuroprotective effects (156). These findings, however, have not always been supported in controlled trials (157). Often, apparently compelling evidence from epidemiological studies does not translate to clinical trials. The reasons for this are not known but potentially include nutrition component interactions with various genetic, dispositional and environmental factors and, of course, the microbiome. Again, while animal models have shown great promise for central effects of modifying the gut microbiota, human trials have been less convincing [e.g., (158)], particularly in normal subjects (non-clinical samples).

Nevertheless, there may be promise for a link between nutritional and psychiatric status. The growing recognition of the role of nutrition in psychiatric disorders was marked by a 2015 position paper in Lancet Psychiatry (159). There is good evidence for e.g., the Mediterranean diet improving mood supported by small clinical trials in non-clinical (160) and depressed cohorts (161). But large mechanistic studies are needed.

Moving forward, nutritional neuroscience will need to better characterize individuals to understand the mechanisms underlying nutrition-brain effects. This will help to understand the enigmatic issue underlying responders and non-responders in clinical trials.

Beyond the science, the future landscape of funding for nutritional neuroscience is relatively promising. The fact that there has been no new “blockbuster” pharmaceutical for the brain this century has led to industry-focused initiatives to develop functional foods for brain health. Organizations like the International Life Sciences Institute (ILSI) Mental Performance taskforce links academia with industry to ensure that claims for links between nutrition and function are evidence-based.

### Intermittent Fasting or Time-Restricted Feeding

(Ellen Blaak)

Recently, there has been an increased interest in identifying alternative dietary strategies for body weight management or improving metabolic health that involved restricting energy to certain periods of the day or prolonging the fasting period between meals. Intermittent fasting (IF) has been shown to be effective in improving cardiometabolic health in several rodent models, ranging from insulin sensitivity and ectopic fat accumulation to hard end point such as stroke and diabetes incidence (162, 163). Prolonged fasting elicits evolutionarily-conserved adaptive cellular responses that are integrated between and within organs in a manner that improves glucose regulation, increases stress resistance, and suppresses inflammation. During fasting, cells activate pathways that enhance intrinsic defenses against oxidative and metabolic stress and those that remove or repair damaged molecules. Additionally, the metabolic switch from the use of glucose as a fuel source to the use of fatty acids and ketone bodies results in a reduced respiratory-exchange ratio (the ratio of carbon dioxide produced to oxygen consumed), indicating the greater metabolic flexibility and efficiency of energy production from fatty acids and ketone bodies. Metabolic flexibility has been strongly associated with metabolic health and insulin sensitivity in humans (164).

Time-restricted feeding (TRF), a relatively new form of IF, is the only eating pattern that does not require calorie reduction and has been shown to improve insulin sensitivity and metabolic health independent of weight changes. TRF is based on circadian biology to allow the body a true daily fasting period in which only water (or non-caloric drinks containing no caffeine or artificial sweeteners) is allowed and aims to maintain a consistent cycle of feeding and fasting to maintain support robust circadian rhythms (165). Studies in rodents, using feeding windows of 3–10 h, report that TRF reduces body weight, increases energy expenditure, improves glycaemic control, lowers insulin levels, reduces hepatic fat, prevents hyperlipidemia, and improves inflammatory markers as compared to eating throughout the day. The molecular mechanisms responsible for the effects of altered meal patterns on metabolic health appears to be related, at least in part, to the synchronization between the time of fasting feeding and the circadian rhythm, including the timely expression of clock-controlled genes, especially those encompassing enzymes and regulatory molecules that mediate physiological and metabolic functions (165). Additionally, the microbiome may be strongly affected by circadian rhythms. Both behavioral and genetically induced circadian disruption have also been shown to decrease the taxonomic diversity and induce intestinal dysbiosis (166, 167). However, enforced feeding-fasting patterns can restore some of these oscillations (168).

Data from human trials suggest that TRF may have similar benefits as in rodents: TRF can reduce body weight or body fat, improve insulin sensitivity, reduce glucose and/or insulin levels, lower blood pressure, improve lipid profiles, and reduce markers of inflammation and oxidative stress (169–171). Additionally, there is potential for humans to adopt TRF as a lifestyle strategy in order to improve metabolic health. Longitudinal monitoring of human eating habits over several days has shown that 50% of the people eat within a time window of over 15 h (171). Moreover, only around 10% of adults maintain a ≥12 h window of fasting.

A barrier to participation in a TRF intervention studies for adults may be, as suggested previously, that TRF may interfere with evening social eating and drinking activities (172). However, in all TRF studies in which negative effects were reported, the timing of the TRF was pre-determined for the participants. Taking an individuals' schedule and personal preferences into account and letting them choose their own TRF interval may greatly improve adherence and efficacy of TRF as well for reducing its adverse effects. The latter strategy has recently been shown to be effective in patients with the metabolic syndrome (170). Overall, sustaining consistent daily rhythms in feeding and fasting may improve metabolic flexibility and molecular rhythms in relevant pathways, thereby preventing disease and improve prognosis. Additional studies are needed to test this further and integrate findings on a healthy diet composition with controlled meal size and patterns with periods of fasting.

### Infancy and the Programming of Human Phenotypic Elasticity

(Bruce German)

The complex processes of early growth and development in infants are the most important targets of nutrition research, yet poorly studied. It is still being established that breast feeding and early nutrition have immediate benefits to various aspects of acute infant health (173, 174). Yet, early dietary quality has far more consequence. Recent studies highlight that diet during infancy has a profound role in influencing long term development of life long phenotype of individuals, now termed programming (175). The urgency to know how these complex processes function as the means to control them is several-fold. The potential of each individual can only be achieved by understanding the effects of earliest diets. Infants born prematurely are increasing alarmingly around the world, and we don't know how to feed them (176). Also, since early diets have consequences to lifelong phenotype, we cannot manage health of adults without understanding what early diet has done to them. Yet, we still do not know what those effects are, even for infants.

The human infant is born relatively naïve, effectively sterile, with an undeveloped immune system, and an anatomical structure that is virtually unable to achieve coordinated movement. Immediately its environment, including the consumption of milk guides the growth, development, maturation and performance of all tissues, structures and processes. Not surprisingly, the paths taken in that development persist throughout life. We need to know how. The challenges to building this knowledge are daunting but there are clear paths ahead for all of life sciences to participate: mechanistic targets of programming, milk's actions on infants and innovative clinical studies. It is equally vital to know the role of maternal diets on lactation, milk composition, and long-term infant outcomes (177).

Mechanistic research is drawing from all of biology to discover the mechanisms by which early environment produces persistent responses in animals. Epigenetic modifications of DNA, histone modifications and chromosome structures all persist in cells after division (178, 179). Tissue hypo and hyper-trophy can themselves persist and diverse metabolic consequences also persist through generations of turnover of the cells that make them up (180). Although the subject is still being actively researched, the various microbial communities within and on infants over the first 3 years of life appear to persist throughout life (181). The myriad consequences of the simple outcome variable: microbial diversity, attest to the importance of early microbiome development (182). The flavor preferences for foods are largely based upon a personal acquisition of memory-based mapping of the olfactory epithelia that takes place in part, even before solid foods through flavorings in milk. Ongoing studies on the mechanisms by which biological imprinting occurs in human infants should be a priority for future nutrition research.

In many ways breast milk is the most illuminating of the paths to understanding how diet acts on acute and long term health (183). Unfortunately, scientists are still struggling with the complexity of determining the simple composition of breast milk throughout lactation and across human maternal diversity (174, 184). The big job of annotating those components for their functions is an aspirational goal for all of life science in part because of the breadth of discoveries to date that attest to the “genius” of lactation evolution. A view of the discoveries related to the macronutrients of milk make a compelling case for breast milk as the gold mine of life science research (185). Protein is not just providing amino acids. Proteomics is revealing multiple targets of intact proteins within the infant (186). Intact proteins guide immune development, gut maturation, pathogen protection, even neurological development (187). Now the implications of specific proteases within milk that activate within the infant (188) reveal a new dimension of milk's bioactive repertoire: milk is a bioactive peptide delivery system (189). The lipids of milk provide substrates, fuels and a diverse signaling system (190). Research has also discovered that digestive reactions within the infant yield an ensemble of hydrolysis products that spontaneously self-associate into complex three-dimensional lipid phases that enhance the absorption of fat soluble nutrients from milk including flavors from the maternal diet (191, 192). Perhaps the most illuminating studies on milk's targets of long-term actions are the complex, undigestible oligosaccharides (193). Human milk oligosaccharides (HMOs) have multiple actions but the most surprising is that they selectively feed specific strains of bacteria and guide overall microbiome metabolism (194). This uniquely diet-managed microbiome provides a breadth of benefits to the infant, lowering inflammation, enhancing vaccine response, pathogen load, fueling, nourishing, and guiding development (195–199). Thus, research to date on milk highlights both the components of diet and their targets of action, both acute and long-term programming.

A central objective of nutrition research going forward is to establish clinical evidence in support of the mechanisms, molecular effectors and health outcomes of the lifelong consequences of early diet. For prospective clinical trials, following infants from birth to death is obviously daunting. However, the challenges to acquiring clinical evidence to understand the efficacy of diet to long term get even worse. Humans are diverse, as is their health. Some of that diversity is achieved by early diets and their persistent effects, but not all. Genetic differences among humans are relatively easy to understand because they are constant. Phenotypic elasticity and its determinants are not so easy to demonstrate because they are variable. In principle, biological imprinting of phenotype is a process to improve an individual's response to an environment. That is its biological value, but its experimental complexity. Thus, the effects of imprinting are “learned” responses to specific inputs. To understand the consequences of biological programming, we have to be able to evaluate the consequences of early diets, not on a fixed phenotype, but on the ability of those individuals to respond successfully, or not, to an environment (200). Clinical nutrition research will be tested by this challenge. Nonetheless, it is an imperative. The field cannot afford another generation of underpowered studies that fail to capture the importance of diet until it is too late.

The world needs large prospective trials inspired by the Framingham, Massachusetts approach to heart disease (201). But we must learn lessons from that study as well. It is critical to capture diet in detail, it is critical to identify and follow proposed mechanisms of programming action in infants at the outset, it is critical to measure subjects accurately and regularly to capture the metadata that inform further hypothesis generation. Finally, it is the responsibility of the nutrition field to maintain a vision. Research that we undertake today will help future generations of humans, inform agricultural processes in our descendants' future and leave a body of knowledge that the scientists who start it will not necessarily benefit from themselves. What better legacy for a field of science?

### Clinical Nutrition at a Crossroads

(Maurizio Muscaritoli)

Human Nutrition encompasses an extremely broad range of medical, social, commercial, and ethical domains and thus represents a wide, interdisciplinary scientific and cultural discipline. Therefore, unlike commonly felt and believed, Human Nutrition represents an intrinsically complex topic. Consensus exists that three main domains can be identified within Human Nutrition, namely, Basic Nutrition, Applied Nutrition, and Clinical Nutrition (202). These three domains have their own cultural and scientific identity, specific aims, and are clearly corresponding to professional skills. On the other hand, while being distinguished, these three domains *are and must be* closely connected and integrated both for academic training and professional activity. However, the gray line separating the purely physiological and cultural aspects from the specifically medical domains of Human Nutrition may appear extremely thin, partly explaining why confusion still exists about skills and competences in nutrition and why the training offered today is still qualitatively and quantitatively inappropriate to target the different professionals involved in the field of Human Nutrition (203, 204).

Among Human Nutrition domains, Clinical Nutrition can be defined as the medical discipline focusing on assessing, preventing, diagnosing, and treating malnutrition (over- and undernutrition, selective deficits of nutrients) related to acute and chronic diseases at all ages (202, 205). Clinical Nutrition aims at studying, preventing and treating the metabolic and body composition changes occurring in individuals at risk of nutritional impairment. Clinical Nutrition employs validated strategies for the evaluation of nutritional status, nutritional therapy and rehabilitation, behavioral and pharmacological approaches, such as dietary intervention for specific pathologies, artificial (i.e., enteral or parenteral) nutrition, or selective supplementation with food for special medical purposes (FSMPs) or specific nutrients (205). Assessment of body composition with sophisticated techniques such as DEXA, bioimpedance analysis, computerized tomography, magnetic resonance, or ultrasonography (206) has progressively become an integral part of nutritional assessment and a peculiar component of the knowledge in the field of Clinical Nutrition. This area of clinical medicine has undergone extraordinary developments and has seen significant advances during the last few years, clearly showing the fundamental role played by body compositional changes on quality of life, morbidity, and mortality of both acutely and chronically ill patients. Besides, the role of specific nutrients (milk proteins, amino acid derivatives, etc.) in the prevention and treatment of sarcopenia (i.e., the progressive loss of muscle mass and function occurring with age or as a consequence of disease) has been elegantly and soundly demonstrated by Clinical Nutrition trials. Clinical Nutrition has substantially contributed to the advancements in the understanding of mechanisms underlying disease-related anorexia and reduced food intake, in unraveling the epigenetic regulation of gene expression by food or specific nutritional substrates and, last but not least, in recognizing malnutrition (undernutrition) as a crucial component of frailty in older adults, which is of paramount clinical relevance.

The SARS-CoV-2 pandemic is clearly showing us that older age, frailty and chronic diseases – commonly associated with undernutrition – may negatively impact on severity of disease, prognosis and survival of COVID-19 patients. In these patients, critical illness and its treatments may be associated with significant changes in nutritional status, body composition and severe disability, definitely highlighting the role of nutrition therapy as a crucial component of the treatment of acute, chronic, and complex diseases including COVID-19 (207). On the other side, the current epidemiological emergency has promoted the generation and diffusion of fake news (208) on the possible “nutritional remedies” to prevent or improve the prognosis of COVID-19 - thus exposing the general population to further health risks, which are hardly defused due to insufficient awareness about Clinical Nutrition within the medical community and the general population.

Although representing one of Human Nutrition's domains, Clinical Nutrition should not be understood as being a sub-discipline of Human Nutrition, but rather as an autonomous discipline with its own objectives, methods and specific domain of knowledge, and as such, it should become a medical specialty similarly to cardiology, gastroenterology, endocrinology, etc. (209, 210). Consistently, undergraduate Clinical Nutrition teaching should be improved and medical students should learn about human nutrition in the first years of a medical curriculum, and they should consolidate the main concepts of Clinical Nutrition during the last 2–3 years. Irrespective of the chosen modality of teaching implementation, this process, which now is progressively recognized as necessary (208) would be accelerated by the recognition of Clinical Nutrition as a well-defined, independent, autonomous – albeit transversal to other medical specialties – discipline. Moreover, the concepts pertaining and belonging to Clinical Nutrition should be taught in medical schools.

A common effort is needed by the scientific community, academic and healthcare institutions, scientific societies, patients' associations, in order to accelerate this process which would ultimately set Clinical Nutrition knowledge free from being dispersed among different “main” specialties (e.g., gastroenterology, pediatrics, internal medicine, endocrinology, …), while converging in one single, “self-standing” medical specialty.

Clinical Nutrition is at a crossroads. It is our moral, scientific and academic commitment to make it proceed in the right direction, in the interest of those thousands of patients in whom anorexia, malnutrition, sarcopenia, metabolic abnormalities related to underlying diseases remain not only untreated, but even largely, and dramatically undiagnosed.

## Sustainability

### Attaining Sustainable Food Systems

(Elliot M. Berry, Barbara Burlingame, Mauro Serafini)

Since the adoption of Sustainable Development Goals in 2016, there have been a number of global, regional, and national reports concerning food systems, the environment and sustainability, which suggest new ways of prioritizing routes toward sustainable food systems in the context of Agenda 2030 (211–217). A consensus has emerged that a systems approach is necessary to address complexity and consider both upstream and downstream effects of making changes. All reports advocate for improved governance and integrated actions, moving away from the developed vs. developing country approaches, and embracing multisectoral and transdisciplinary policies and actions, and emphasizing the need to articulate local experiences along with global approaches. The Right to Food is also highlighted as the overarching, fundamental principle.

A Sustainable Food System “*is a food system that ensures Food Security and Nutrition (FSN) for all in such a way that the economic, social, and environmental bases to generate FSN of future generations are not compromised*” (215). According to the Global and Environmental Change and Food Systems project (http://www.gecafs.org), Food Systems encompass: (1) activities related to the production, processing, distribution, preparation, and consumption of food and (2) The outcomes of these activities contribute to the dimensions of food security: Food availability, with elements related to production, distribution, and exchange; Food access – affordability, allocation & preference; Food utilization – nutritional value, social value and food safety; and Stability - ability to ensure food security in the event of sudden shocks. These outcomes also contribute to environmental and other securities (e.g., income, health).

Further, during the past decade, a fifth concept, Sustainability, has been added as the long-term time dimension involved in food security (214). This brings a more comprehensive and holistic approach to sustainable food systems which considers drivers—environment, geopolitics, demographics, policy regulations, socio-cultural-economic factors, science and technology, and infrastructure. The outcomes, similarly, involve many dimensions—environment, food security and nutrition, health and socio-cultural-economic aspects (215). And even more recently, a sixth concept, Agency, has been proposed to emphasize the role of individuals and groups to act independently and make free choices about what they eat and how that food is produced, processed, and distributed (216). Interactions between and within bio-geophysical and human environments influence both the activities and the outcomes. Implicit in the above is the need for Sustainable diets (214, 218).

There is the recognition that practical attainment of such essential goals requires multi-stakeholder involvement and expansion of food systems to deal with geo-political challenges posed by the progressive urbanization of the world population and man-made and natural disasters. This means considering representatives from agriculture (farmers and global business), nutrition, health, emergency preparedness, economics, education, academia, food industry, retailers, restaurant chains, street vendors, NGOs and civil society (consumers), law, policy makers (government, local authorities), and more. In particular to note that the food industry should be involved in the process with incentives (or legislating) for improved formulations and disincentives (e.g., taxing) for ultra-processed foods and their advertising, especially to children.

These issues are further complicated by the disconnect between urban and rural policies. The current geo-political climate would indicate a move away from globalization to territorial approaches (Urban Rural Link, Fostering Terit Perspec). Therefore, discussions on food systems need more emphasis on the socio-cultural aspects of the sustainability agenda where food and nutrition is a major cultural transmission link.

Moving toward integrated food policies and sustainable food systems will only occur if the democratic deficits in food systems and power disparities are addressed. By shifting the focus from agriculture (and other sectoral policy areas) to food, a wider range of stakeholders can be meaningfully involved in designing and assessing policies and actions. Recognition that overweight and obesity are as much an agriculture and environment sector problem as a health sector problem; and that thriving ecosystems are as much a health sector issue as an agriculture and environment sector issue will allow imbalanced relations and path dependencies to be more effectively addressed. In the process, decision-making can be reclaimed from influential lobbies, and new priorities and coalitions of interest can emerge (219).

Cities are becoming key players in encouraging more sustainable food systems. More than 200 mayors have signed the Milan Urban Food Policy Pact (220) and are generating and exchanging significant local experiences, which can be shared and fed back into more coherent national and global policies. They are promoting urban agriculture and healthier food environments, while also improving parks, safe walkways and bicycle paths to facilitate physical exercise (221).

Biodiversity for food and nutrition is indispensable to food security, sustainable development, and the supply of many vital ecosystem services. Research on food and agricultural systems needs to become more multidisciplinary, more participatory and more focused on interactions between different components of biodiversity. Local fresh fruits, vegetables, and other plant foods, including neglected and under-utilized species and varieties, are often of higher nutritional quality than imported foods produced with industrial farming methods. The use of environmentally sustainable - in particular biodiversity-friendly - practices is increasing and agri-ecology (222) is receiving renewed interest as a viable alternative to present agricultural practices. Evidence is accumulating on multi-purpose strategies and local practice through active research programs. Agri-ecology and nutrition-driven food systems research should be a priority with methodologies from multiple disciplines (223). It is important to understand determinants and interactions (including biological, psychological, and social) at the local level, adopt a territorial (in particular bioregional) approach and develop multi-purpose strategies. Finally, and most importantly, the impact of climate change on food systems and food security will have to be addressed more aggressively in the global context (224, 225).

Concrete Action Proposals (219). The following strategies, which are consistent with many of the SDG targets, may accelerate the progress toward more sustainable food systems in the future:

1. Promote collaboration between nutritionists and environmentalists in relevant bioregions by (i) encouraging biodiversity-friendly practices for food and agriculture and enhancing their contribution to ecosystem services and by (ii) focusing on hotspot areas and food security of the most vulnerable population groups (no one to be left behind)
2. Partner with cities and local governments to generate practice-based evidence and ensure synergy with national and global processes;
3. Encourage teamwork between scientists, policy makers, politicians, and practitioners;
4. Enable consumers to adopt sustainable diets and engage with civil society and the media to ensure that sustainable food systems become priorities on the political agenda;
5. Enforce accountability for private sector and policy makers to deliver sustainable development.

Each country should endeavor to follow the policies and agendas best suited to their circumstances. Political will and grass root support from the young are essential to see that future generations and their politicians adhere to policies supporting sustainable food systems and implementing Agenda 2030.

## Food Processing and Manufacturing

### Future Food Technologies

(Dietrich Knorr)

The purpose of this contribution is to provide a short overview of needs and priorities for a necessary change of key food related research and development areas.

#### Worldwide Multidisciplinary Interaction Along an Integrated Food Chain

The recent Covid-19 based disruption in current global food delivery chains, leading to poverty, hunger, and further inequalities among humans (226); the sheer complexity of our food with over 26,000 distinct, definable biochemicals present (227); the uncertainty in human nutrition research (228); the ongoing issues with consumer trust and food products and processing (229); the need for an holistic and transparent approach toward food processing, as well as the current debate on ultra-processed foods (230) are clear indications for the need of new strategies and change.

Consequently, a worldwide collaboration of all players along the food value chain is needed, especially between agriculture, food science, nutrition, and consumer science, to develop a future agenda in which transdisciplinary research becomes the norm (231).

Lillford and Hermansson convincingly demonstrate the lack of interaction between agriculture and nutrition with food science and developed a series of mission statements requiring multidisciplinary collaboration (232). These interactions should not only include a rational debate among scientists but also the willingness of a better appreciation of each other's expertise. Successful examples for such an approach is the worldwide collaboration of electrical engineering, biotechnology, environmental science, medicine, and food science around pulsed electric field technologies (233) and the existence and activities of Frontiers in Nutrition: Nutrition and Food Science Technology.

#### Strengthening Trust in Food and Nutrition Science

A paradigm change was initiated when developing the PAN concept referring to the need for tailor made foods that encompass all consumer PREFERENCES, ACCEPTANCE, and nutritional NEEDS. This necessitates a complete re-design of the way food is produced and suggests that rather than adapting food raw materials to the requirements of emerging technologies, gentle, flexible, and scalable technologies need to be used and developed to be adaptable to preferences, acceptance, and needs of consumers. The originators of this concept (234) also discussed *reverse engineering* because product development needs to be modulated back through the food chain from the consumers to raw materials.

This concept goes hand in hand with another paradigm change developed (234), which relates to *process-structure-function/property* relationship of foods. Here, targeted processes are developed and used to create tailor-made food structures, which will then deliver specific and desirable food functions and properties.

The challenges related to these concepts are the need for in-depth understanding of the food processing technologies applied as well as the accumulation of related data about mechanisms and kinetics of food constituents, structures and constituents, and subsequent food behavior and interactions.

Consumer science will need to deliver reliable data on consumer preferences and acceptances and nutritional sciences are required to accumulate relevant data for consumer needs (234, 235).

This reverse engineering concept was originally directed toward food processing and consumer needs but now needs to be expanded to include also the production of food raw materials toward the Preference, Acceptance, and Needs of the consumers.

The emerging food technologies, aiming for gentle, green and sustainable processing, and related benefits (229, 236–238) offer new routes of processing (e.g., combination processes conventional-emerging or emerging-emerging technologies) as well as new possibilities to use new raw materials such as insects (239), new proteins (240), or microbial and cell culture biomass (241, 242) and intelligent packaging (243) (see also below).

As for nutrition science, there have been pleas for long term randomized diet intervention trials (244, 245) and more consistent and reliable information (246, 247).

One approach that has been taken by a multidisciplinary collaboration around pulsed electric fields as an example for an emerging technology, was the development of guidelines for experimental research designs and data reporting (248). These concepts could be expanded to other technologies and disciplines.

#### Nutritional Aspects of Emerging Technologies

Impressive successes and resulting industrial developments have been achieved as a result of research activities on emerging technologies such as high hydrostatic pressure and pulsed electric fields since their onset in the 1980's (233, 249, 250). Additional new technologies have been developed including pulsed light, ultra-violet and infra-red processes, ohmic heating, cold atmospheric plasma, and 3D/4D printing.

However, limited data exist regarding nutritional evaluations of products subjected to these processes (236, 250) having different modes of action to conventional ones.

Carmody, Weintraub, and Wrangham have already demonstrated the distinct energetic differences of foods subjected to thermal processing (cooking) or non-thermal processing (pounding) (251). Further, differences in energy intake rates due to oral processing have also been shown recently (252) adding an additional layer of complexity for well-controlled human feeding studies.

Increased attention needs to be given to the interaction of emerging technologies and new raw material development and nutritional evaluations.

#### Feeding an Urban Planet

The need for radical changes in current food systems to be able to feed the two thirds of the planet's population expected to be living in mega-cities (20+ million) within the next 20 to 30 years has been highlighted previously. Recommendations and actions included urban food production, processing concepts; food and water management practices; smart/precision food preparation and delivery concepts; appropriate and zero waste technologies; intelligent food packaging; and consumer science and interactions. Some activities have been carried out in urban agriculture/horticulture (253), but relevant concepts for appropriate storage, processing (e.g., robust, flexible, scalable, and modular systems) preparation or delivery are still missing. This is especially challenging since a high percentage of urban citizens will eat away from home (254).

#### Resilience of Food Systems

The severe impact of COVID-19 on the 17 UN Sustainable Development Goals also exposes the fragility of these goals with the one of good health being the most obvious casualty (255), is another indication for the need of radical changes within our food systems.

The term sustainability has been used excessively without a clear understanding of its meaning and a severe lack of agreement upon indicators of food system sustainability (256).

The interactions between food processing and sustainability and the improvement thereof have been reported extensively (257–260). The need to understand the fundamental character of interaction between nature and society (261, 262) also indicates the necessary interaction between food systems and climate change requiring additional future awareness and actions.

Within this context the role of food processing in food and nutrition security (including food safety and traceability) also requires attention (240, 263, 264). This needs to play a role in any future scientific debate at an interdisciplinary level with the most pressing ones being, resource management; sustainable processing and improved delivery; consumer behavior and trust as well as sustainability integration into the entire food value chain.

#### Ultra-Processed Foods

The main reason for the (re-)emergence of emerging technologies was based on consumer and food processors' demands for gentler (with the German term “*schonend*” being a more accurate description), greener, less chemical based, less energy input (especially heat) technologies. This resulted in the first industrial, high hydrostatic pressure processed products in 1990 with currently about 520 installations worldwide.

Approximately 20 years later a new term emerged for mainly energy dense and cheap products (previously called fast food, junk food, convenience food…) as “ultra-processed” foods (265). Additional publication from the same group presented the NOVA food classification concept (265–267) consisting of four groups: unprocessed or minimally processed foods, processed culinary ingredients, processed foods, and ultra-processed foods. Ultra-processed foods have been defined as industrial formulations typically with five or more ingredients, mainly referring to sugar, oils, fats, salt, antioxidants, stabilizers, and preservatives.

The NOVA classification system has been considered as a rather simple and crude system of classifying food into categories on the basis of their degree of processing from the nutritional point of view (268). It has been seen in stark contrast to the many already existing and more sophisticated systems such as Foodex, EPIC or Langual™ Subsequently, it was further demonstrated how the definition of ultra-processed foods has been changed by the initiator of the NOVA concept between 2009 and 2017 (269).

The fact that the NOVA definition does not refer to processing or unit operations used for processing and preparation of foods has been discussed previously (230). From the food process engineering end, one has to conclude that the selection of the term ultra-processed does not relate to food processing *per se* but is based on a misunderstanding (or lack of understanding) of the complexity of food materials and food processes. Further, it was cautioned that the requested avoidance of specific food products termed as ultra-processed could lead to deficiencies in micronutrients and fiber (270, 271).

In agreement with Gibney (268), Knorr and Watzke (230) argued that a rational and worldwide scientific debate is needed especially at the interface of food science and nutrition as well as a forceful paradigm shift in food research addressing coherently all the unresolved questions in food science and nutrition.

Key recommendations and actions:

1. Worldwide compendium and description of all unit operations for food processing as well as tools, machines, and equipment used for food processing and preparation including traditional ones
2. Re-evaluation of existing processes regarding energy efficiency, sustainability, nutrient change/retention/increase/losses
3. Development of transparent processing chains
4. Food science and nutrition partnerships to design nutritional evaluation experiments for products from new processes
5. Collaborative studies on food structures generated by targeted food processing to deliver tailor made food for specific needs and requirements of different consumer groups as well as regional needs
6. Collective emphasis on regaining consumer trust via consistent, reliable, and transparent consumer information and education
7. Global scientific and rational debate between and among agriculture, food nutrition, and consumer scientists regarding solutions for unanswered question to face the challenges of our food systems.

### Healthy and Sustainable Protein

(Johannes le Coutre, Mauro Serafini)

Global protein consumption rose by 40% between 2000 and 2018 with over 50% of this increase being driven by Asia (272). Global meat consumption is expected to increase by 76% in 2050 (273). This increased demand requires to scale production significantly. Given constraints on land and water availability and the impact of meat production on climate change, increased efficiencies in production practices and improvements throughout the wider food chain will be critical (274). Advances in novel technologies such as cellular agriculture or also the improvement in sustainable traditions such as the consumption of insects (Entomophagy) might play an important role for balanced solutions in decades to come.

Animal farming accounts for 14.5% of Greenhouse gas emissions from human activity while in comparison aviation is responsible for only 2%. The effect is so big because of the number of animals and because of the associated feed production. According to the Food and Agriculture Organization of the United Nations (FAO), over 74 billion terrestrial animals were slaughtered in 2016. In the US, almost 70% of the grain is grown for animal feed. All international organizations including IPCC, FAO, OECD, NGOs, and EAT Lancet commission call for reducing meat consumption in order to mitigate climate change. This awareness is beginning to change food preferences - a trend that will just increase.

Breaking the link between animals and meat is a solution to this problem.

Over the past decade significant progress has been made in trying to utilize technologies developed in the medical and in the biomedical scientific areas to grow food from the cellular level. This approach, also known as Cellular Agriculture, holds the promise to relieve agriculture from the burden of not being sustainable. Specific tissues of food, be it from vegetables or from meat can be produced at the cellular level.

The production of cultivated meat will require only a fraction of the land area that is used for producing the same mass of conventionally produced meat. In conventional meat production, the potential for reducing GHG emissions is limited because most of the emissions are due to methane from manure and ruminants' enteric fermentation and nitrous oxide from soil.

For the production of cultivated meat, a small biopsy is obtained from the live animal and the material is subjected to a number of biological steps involving proliferation and differentiation. Scaling of the material at an acceptable cost remains still a key challenge in order to obtain sufficient amounts of edible material (275). Another challenge will be to create the nutritional and organoleptic features of an animal piece of meat.

Domestication by humans of plants and animals took millennia to result in our current green and livestock dependent agriculture. It might take only decades to embark on an initial wave of second domestication to harness the benefits of micro-organisms via cellular agriculture (276).

Initially, cellular agriculture will provide cleaner products, which will be slaughter-free from the beginning. Products will be costly, and they will be catering to a niche population of consumers only, much of whom will be early adopters. Those initial products will be linked to a reduction of microbial outbreaks, antibiotic exposure and to a reduction of methane, and carbon dioxide emissions through livestock.

Edible animal protein can also be sourced through Entomophagy, which has accompanied human history through centuries playing a significant role in cultural and religious practices. A hallmark of edible insects is their low ecological cost, which recently sparked renewed global interest through the need to obtain drastic reduction of the ecological impact of food from animal sources. On the basis of their tissue composition, edible insects are characterized by an excellent pattern of proteins, polyunsaturated fatty acids, vitamins, and minerals. Moreover, recent studies provide evidence about their remarkable content of antioxidants (277), an inhibitory activity of lipoxygenase and cyclooxygenase-2 (278) and anti-inflammatory actions (279), confirming ancient use in traditional medicines from indigenous populations (280). Research on the functional role of edible insects represents an innovative and promising research field for coming years. Considering that insects are already part of the world's common diet for at least 2 billion people and that there are one million species, they hold the promise to represent a significant nutritional resource.

### Food Integrity and Food Safety

(Michael Rychlik)

The Novel Food Systems outlined above (e.g., Sustainable Food systems, Cellular Agriculture) open a perspective to the agri-food “community” to supply foods of high integrity, i.e., being sound, nutritious, healthy, tasty, safe, authentic, traceable, as well as ethically, safe, environment-friendly, and sustainably produced (281).

However, verification of food integrity is still challenging and the issue of fraudulent foods has been mentioned already in our last Field Grand Challenge (1). Food fraud can be fought with two orthogonal approaches, namely comprehensive food analyses and tracing methodologies.

In this regard, comprehensive food analysis will target the “*foodome*,” which recently has been defined as the “collection of all compounds present at a given time in an investigated food sample and/or in a biological system interacting with the investigated food” (282). Although the *foodome* still mainly consists of “dark matter,” there are perspectives that different aspects of it may be uncovered in the next two decades if not before (283).

Nevertheless, food analysis of the future increasingly has to include chemometry and, for validation of the results, also metrology, i.e., “the science of measurement, embracing both experimental and theoretical determinations at any level of uncertainty in any field of science and technology” (284). With respect to metrology, METROFOOD-RI, the pan-European *Research* Infrastructure for promoting Metrology in Food and Nutrition aims to promote scientific excellence in the field of food quality and safety (281). METROFOOD-RI recently has been approved by the ESFRI (European Strategy Forum on Research Infrastructures) and started its preparatory phase to become operational soon.

Food safety as one important part of its integrity will have to cover, on the one hand, emerging contaminants such as mineral oil hydrocarbons (MOH), polyfluorinated alkyl substances (PFAS), pyrrolizidine alkaloids (PA) (285), emerging mycotoxins produced by *Alternaria* fungi (286) and the very scarcely understood complex of microplastics and nanomaterials. On the other hand, microbial safety is still crucial for food safety, with *Listeria, Salmonella*, and the Norovirus among others causing severe food poisoning worldwide.

## Summary and Outlook

Taken together, with the editorial board of Frontiers in Nutrition, we see a succinct and actionable number of key goals and challenges to improve our global food system.

Engagement Goals:

- Improved scientific interactions among all stakeholders within global food systems to improve intra- and inter-disciplinary collaboration and understanding for improved consumer trust.
- Terms and disciplines such as Functional Food and Clinical Nutrition matured into well-defined, autonomous, transversal concepts, and should be included in medical school undergraduate education.
- Genes have not changed over the past decades and the obesity pandemic is largely caused by a toxic, obesogenic environment, which affects in particular lower socio-economic groups. Prolonged positive energy balance leading to overweight is a behavioral risk factor that may develop into disease.
- Obesity prevention and management require a multi-level approach involving National policy; Food, Education and Medical systems; Public Health, Local Authorities; Community involvement; and Parental and Individual responsibilities.
- Collaboration is needed between nutritionists and environmentalists to encourage biodiversity-friendly practices for sustainable diets and agriculture and to focus on the most vulnerable food insecure population groups.
- Engagement with policy makers, civil society, and the media is necessary to ensure that sustainable food systems are priorities on the political agenda.

Technology Goals:

- Strengthen human-based evidence and focus on real life settings in dietary intervention studies.
- Functional foods to take part in the restoration and optimization of health promoting gut microbiota.
- Nutrition to become involved in therapeutic and preventive strategies to promote immunological tolerance in the control of chronic inflammatory disease.
- Successfully address the sustainable protein challenge.
- Target pathways at the intersection of immunity and metabolism to induce conserved regulatory mechanisms that provide durable clinical response and remission in infectious and autoimmune diseases.
- Focus on human systems biology to improve the quality of research in the field of sports nutrition, enhance interpretation of the body's complex response to exercise stress and nutritional interventions, and expand the capacity for adapting nutritional recommendations at the individual level.
- Deliver prospective evidence to support the clinical efficacy, cost-effectiveness, and additional benefits of personalized or subgroup-based approaches beyond traditional nutrition intervention approaches.
- Provide additional studies to investigate how daily rhythms in feeding and fasting may improve metabolic flexibility and health and integrate findings on a healthy diet composition with controlled meal size and patterns with periods of fasting.
- Establish how early diet influences the lifelong phenotypes of humans.

Evidently, food and nutrition science do experience a renaissance for a number of years. This change in recognition is built upon insights from epidemiological, clinical, and physiological studies as well as technological, environmental, and policy-based advancements alike.

Personal and planetary health are taking center stage in this development and possibly the past 5 years have seen some alignment in cross sectoral efforts because of the SDGs. Disturbingly, Covid-19 demonstrated a worrisome vulnerability in our societies and it appear to be the food systems involved, which need to be strengthened for the future. Science has a role to play in this endeavor.

## Author Contributions

All authors listed have made a substantial, direct and intellectual contribution to the work, and approved it for publication.

## Conflict of Interest

The authors declare that the research was conducted in the absence of any commercial or financial relationships that could be construed as a potential conflict of interest.
